# Leveraging latent profile analysis to synthesize childhood and adolescent risk factors for suicidal ideation

**DOI:** 10.1371/journal.pone.0272400

**Published:** 2022-08-31

**Authors:** Katherine Sarkisian, Elizabeth Planalp, Carol Van Hulle, H. H. Goldsmith

**Affiliations:** University of Wisconsin–Madison, Madison, WI, United States of America; Temple University, UNITED STATES

## Abstract

Person-centered typologies identified with latent profile analysis can clarify patterns of chronic and acute risk factors for suicidal ideation. We derived five profiles of individuals using cognitive, behavioral, and familial factors relating to suicidal ideation risk factors. Participants (n = 1,142) were assessed at age 8 using the Laboratory Temperament Assessment Battery and mother-reported parenting measures and at age 14 using interviews about clinical symptoms and suicidal ideation. The best-fitting model included five profiles: *typical*, *elevated adolescent symptomology*, *mildly elevated typical*, *low childhood persistence*, *and very low childhood persistence/mixed symptoms*. Participants in the *elevated adolescent symptomology* and *very low childhood persistence/mixed symptoms* profiles were 2.6 and 5.3 times more likely to report suicidal ideation compared with the *typical* profile. Overall, our results underscore how using a person-centered pattern recognition approach and incorporating facets of childhood behavior may enhance conceptualizations of adolescent suicidal ideation risk.

## Introduction

Suicidal ideation is attributable to a confluence of risk factors, making a thorough understanding of its behavioral determinants difficult. However, multivariate, person-centered approaches, like latent profile analysis, can offer useful insight into how constellations of concurrent and distal variables, such as those posited by prominent conceptual models, relate to suicidal ideation. The cognitive theory of suicidal behavior ([1] p. 192) proposes that problem-solving difficulties “[create] a context that is ripe for a host of psychiatric symptoms, hopelessness, and eventually, suicide ideation to emerge.” This conceptual model aligns with our prior findings linking childhood problem-solving persistence [2], low levels of positive parenting (i.e., a predictor of child hopelessness [3, 4]), and maladaptive cognitive tendencies (i.e., inattention, impulsivity, brooding) to adolescent suicidal ideation [5]. However, the variable centered regression analyses that are often used to examine individual suicidal ideation risk factors may not fully reflect the complexities of theoretical models or clinical presentations. Here, we used latent profile analysis to examine typologies that combine cognitive and familial suicidal ideation risk factors with behaviorally measured problem-solving persistence. We contend that this person-centered approach that blends a novel distal correlate with well-established cognitive and familial ones is more reflective of individual processes and may ultimately inform the design of earlier and more individualized interventions to reduce suicide risk.

### Risk factors for adolescent suicidal thoughts and behaviors

In the United States, suicide is the second leading cause of death for individuals between the ages of 10 and 19 [6]. In addition to overall increases in suicide rates, a striking 75 percent increase in rates of death by suicide among adolescent girls highlights the necessity of improving prediction, prevention, and intervention methods [6, 7]. In the past 50 years of research, a number of risk factors ranging from psychopathology to social and biological factors have been evaluated [8]. Risk factors including negative family environments, firearm access, and adverse childhood experiences are well-characterized, but it remains unclear how these risk factors interact to shape short- and long-term risk for suicidality during childhood and adolescence [9–12]. Prediction of suicidal thoughts and behaviors has improved only marginally despite decades of research, and most existing studies use univariate statistical models to predict longer-term suicidality related outcomes [8, 13]. Although these limitations can be attributed to numerous factors, including flaws in study design and a general lack of novel risk factor tests, failure to examine the combined effect of multiple risk factors has been a pervasive issue [8].

Comprehensive risk conceptualizations that reflect constellations of short- and longer-term cognitive, behavioral, and familial risk factors would likely facilitate more effective risk prediction [8]. For instance, in a latent class analysis of psychiatric comorbidities among adults who had made a suicide attempt within the past 24 hours, three clinically useful classes emerged: “major depressive disorder,” “high externalizing disorder,” and “high internalizing and externalizing disorders” [14 p.1]. Although these classes were not differentiated by demographic variables or suicidality severity, both concurrent and distal clinical variables (e.g., binge drinking within the past year, history of receiving outpatient therapy, using an illegal drug in the 24 hours before a suicide attempt) contributed meaningfully to differentiating these risk typologies [14].

Moreover, assessments of concurrent and distal risk factors among children and adolescents need to be conducted using a developmentally appropriate, multi-informant approach. Significant discrepancies between parent and child or adolescent reports of psychopathology symptoms are possible, particularly when assessing internalizing symptoms [15, 16]. Consequently, we focused on adolescents’ self-reported symptomology and combined it with both behaviorally measured and parent-reported variables (i.e., parenting approaches) from childhood, which is consistent with the multi-informant approach that typically yields a “valid, but not redundant” picture of child and adolescent psychopathology and functioning [17 p. 1576].

In addition to risk factors with varying degrees of temporal proximity, transdiagnostic risk factors that may contribute to clinically meaningful typologies are equally important to examine. In a latent profile analysis of lesbian, gay, and bisexual youths, a profile of individuals who slept the least and had elevations in alcohol use, bullying, poor grades, and electronics use were 17 times more likely to make a suicide attempt compared with a profile of “low risk” peers [18]. In a subsample from the longitudinal Wisconsin Twin Project, we found that adolescents who exhibited elevations in brooding, inattention, and impulsivity were significantly more likely to report concurrent suicidal thoughts [5]. Inattention predicted increases in concurrent risk most robustly; therefore, we then evaluated developmental antecedents of inattention by creating an early problem-solving persistence composite that included behaviorally coded effort duration and intensity during problem-solving, mother-reported attentional control, and observer reported persistence [2]. Adolescents who demonstrated low levels of persistence during childhood problem-solving tasks were significantly more likely to report suicidal thoughts, which is consistent with findings of problem-solving difficulties among individuals who exhibit more severe suicidal thoughts and behaviors [2, 19]. This association between low childhood problem-solving persistence and adolescent suicidal ideation parallels a similar study in which low problem-solving effort could be observed reliably among kindergarten-age children [20]. Behavioral manifestations of learned helplessness (e.g., earlier and more pronounced negative effects on mood, decreased hopefulness, negative self-evaluations) were exacerbated by authoritarian parenting practices and appear to be a precursor to a more stable depressive cognitive style [20]. Because parenting practices that may be associated with increased child and adolescent suicidal ideation risk tend to be defined broadly [20, 21], we sought to examine specific facets parenting, including parent encouragement of emotional expression and rational guiding (*i*.*e*., positive feedback, appropriate limit setting) related to subsequent suicidal ideation risk [4]. Experiencing higher levels of both facets of positive parenting during childhood was associated with significantly lower risk for suicidal thoughts during adolescence, which parallels findings regarding the negative effects of neglectful and authoritarian parenting on adolescent suicide risk [4, 21]. All of these non-diagnostic, longitudinal, and/or behavioral predictors represent a departure from traditional concurrent, self-reported risk factors such as depression symptoms and substance use and highlight the utility of exploring novel risk factors [22].

### Implementation of latent profile analysis in our sample

Because previous work indicates that cognitive, behavioral, and familial risk factors individually predict increased risk for concurrent and subsequent adolescent suicidal ideation [2–5, 23, 24], we sought to create more comprehensive typologies to clarify how different combinations of these variables relate to suicidal thoughts. A latent profile analysis approach to adolescent depression [25], which derived five profiles of symptoms that characterized their sample, serves as a prototype of this approach that we can apply to suicide-related factors. Compared with piecewise analysis of individual risk factor variables, risk typologies that characterize patterns of risk factors at the individual level may better inform the design and implementation of comprehensive interventions.

Here, we build upon existing literature and our earlier logistic regression-based analyses, but we pivot from variable centered analyses to person centered analyses. Rather than simply identifying variables that predict suicidal ideation, we focus on behavioral patterns (profiles) of variables in a large sample. We included continuous variables, including positive parenting at age 8, observed problem-solving persistence at age 8, and self-reported inattention and impulsivity, brooding, and depression symptoms at age 14. Because our sample includes measures from assessments during childhood and adolescence, we have a rare opportunity to illustrate the meaningful ways in which latent profile analysis can capture typologies that involve distal and concurrent risk factors.

## Method

### Participants

The longitudinal twin sample was recruited using birth records and was mildly enriched for psychopathology during middle childhood (*i*.*e*., at least one member of the pair scored at least ½ standard deviations above the mean level of depression, anxiety, over-anxiousness, oppositional defiance, aggression, conduct disorder, inattention, or impulsivity, all reported by participants’ parents using the MacArthur Health & Behavior Questionnaire [26, 27]; cotwins of participants above at least one of these cutoffs and twin pairs in which neither member was above these cutoffs were also included [28].

The sample used for analyses (N = 1142) included individuals who had data for at least one of the indicator variables. Of these, 47.5% were female (i.e., sex assigned at birth, as reported by the mother). For participants with non-missing ethnicity data, 93.4% were White (n = 1,054), 3% Black (n = 34), 1.2% Native American (n = 14), and <1% Filipino, Laotian, Korean, Hmong, biracial, or “other race”. Parents reported mean household incomes of $70,001-$80,000. This sample was not selected in any way for suicide risk. The childhood assessments (i.e., mother’s report of positive parenting, in-home temperament assessment that included problem-solving tasks) were completed at mean age 8 years, and the adolescent assessments (i.e., assessment of inattention, brooding, and impulsivity; diagnostic and suicidal ideation assessments), were completed at mean age 14 years.

For validation analysis, we used a subsample of participants (n = 45) who reported suicidal ideation; these participants were significantly more diverse than the full sample in terms of ethnicity, family income, and parents’ marital status. Fifty-five percent of adolescents who reported suicidal thoughts had parents who were not married to each other (*e*.*g*., divorced, widowed), which differs significantly from the rest of the full sample in which approximately 90% of participants’ parents are married to each other [29]. Among individuals with suicidal ideation, 17.5% are Black, 5% are Native American, and 2.5% are Chinese. Informed assent was obtained from all twins included in the study and informed consent was obtained from their parents. The Institutional Review Board at the University of Wisconsin–Madison approved all study procedures.

### Measures

#### Positive parenting

Mothers reported on their parenting practices for children, including encouragement and modeling of emotional expression, and rational guiding, using a shortened version of the Child-Rearing Practices Report [30]. Rational guiding includes behaviors like parents’ use of positive reinforcement and validation. The encouragement of emotional expression composite includes tendencies like fostering curiosity and openness, as well as asking about children’s troubles in a supportive manner. These two parenting measures were significantly correlated (*r* = .45, *p* < .001) and thus averaged.

#### Childhood persistence

Persistence was measured at the childhood assessment using (1) mother-reported attentional control; (2) observed duration and intensity of effort during problem-solving episodes from the childhood version of the Laboratory Temperament Assessment Battery [31]; and (3) trained observers’ ratings of participants’ persistence throughout the temperament assessment battery. The intercorrelations of these three variables averaged .5 and thus we standardized and then averaged them.

#### Inattention and impulsivity

Adolescents reported on their inattentive and impulsive behaviors within one month of the diagnostic interview using the MacArthur Health and Behavior Questionnaire (HBQ). The HBQ assesses physical health and mental health symptoms, adaptation, and impairment [26, 27]. These two items were significantly correlated (r = .73, p < .001) so we combined them into an average score for inattention/impulsiveness for further analyses.

#### Brooding

Adolescents provided self-reports of rumination using the Response Styles Questionnaire [RSQ; 32]. The RSQ contains five items that assess brooding (*e*.*g*., “I think ‘Why do I always react this way?’”) and uses a response scale ranging from 1 (“almost never”) to 4 [“almost always”; 32]. We formed a brooding composite by averaging the five item-level brooding scores and then standardizing this composite.

#### Depression symptoms

Adolescents self-reported depressive symptoms using the structured diagnostic interviews administered by trained interviewers. We used both the Diagnostic Interview Schedule for Children (DISC; Version IV) and the DISC Predictive Scales (DPS); however, these interviews are quite similar, and both were created using DSM-IV criteria and have good reliability and validity [33, 34]. Because we wanted to examine symptom severity, rather than a categorical diagnosis, we created an ordinal variable to equate different symptom scale lengths between the two interviews. Specifically, we recoded participants’ number of depression symptoms into four categories (0 = 0 symptoms; 1 = ≥1 symptom, but less than symptom count equivalent to 74th percentile [1–6 symptoms on DISC; 1 symptom on DPS]; 2 = >74th percentile, but did not meet diagnostic criteria [≥7 symptoms on DISC, but less than diagnostic cutoff; ≥2 symptoms on DPS, but less than diagnostic cutoff; 3 = met diagnostic criteria [diagnostic criteria for the DISC and DPS differ, so we used the validated, predefined clinical cutoff for each]).

#### Suicidal ideation

Suicidal ideation endorsement is used for the validation step. Two items during the depression modules of the DISC and DPS diagnostic interviews described above assessed whether the participant had experienced active suicidal ideation in the past year and whether they had ever made a suicide attempt. Of the 1,142 participants, 46 endorsed ideation, but one did not have any data for the indicator variables used in analyses so was dropped from further analysis. Phone calls with the 46 participants who endorsed ideation were transferred immediately to a clinically trained researcher who conducted a more thorough suicide risk assessment that included questions about suicidal ideation, intent, potential suicide plans, self-harm, prior suicide attempts, recent significant stressors, and social support using an IRB-approved protocol. Clinically trained researchers assessed whether the severity of the participant’s suicidal ideation, intent, and possible suicide plan warranted emergency intervention (i.e., high likelihood of participant making a suicide attempt within 24 hours). Emergency intervention was not required by any of the participants who endorsed suicidal thoughts; thus, our efforts were focused on referrals to appropriate mental health resources, notifying participants’ parents of their suicidal thoughts per the breach of confidentiality statement in our adolescent assent form, and safety planning, including means restriction (e.g., urging families to secure firearms, if relevant).

## Results

Descriptive statistics for the variables are shown in Table 1, along with correlations between study variables. In general, higher levels of depression symptoms correlated positively with brooding (*r* = 0.37, *p*<0.01) and inattention/impulsivity (*r* = 0.35, *p*<0.01). Similarly, inattention/impulsivity correlated positively with brooding (*r* = 0.26, *p*<0.01). Weak, but statistically significant, negative correlations of problem-solving persistence with brooding (*r* = -0.082, *p*<0.01), inattention/impulsivity (*r* = -0.18, *p*<0.01), and depression symptoms (*r* = -0.15, *p*<0.01) were noted. Although these correlations are significant, their small size suggests sufficient conceptual distinctness between problem-solving persistence and symptomology. Notably, positive parenting did not correlate significantly with any of the other variables.

**Table 1:** Means, standard deviations, and bivariate correlations among indicator variables included in latent profile analysis.

	Mean	SD	Range	N	1.	2.	3.	4.
1. Positive Maternal Parenting	5.50	.42	3.5 to 6.00	1116				
2. Childhood Persistence	-.005	.69	-3.16 to 2.89	1118	.02			
3. Adolescent Inattention/Impulsivity	2.74	.86	1.00 to 5.37	1124	-.03	-.18**		
4. Adolescent Brooding	2.07	.63	1.00 to 4.00	1121	.01	-.09**	.27**	
5. Adolescent Depression Symptoms	0.91	.80	0.00 to 3.00	817	-.003	-.14**	.36**	.37**

*Note*. ** = p<0.01 (2-tailed).

### Latent profile derivation and selection

To determine the number of profiles that best fit our data, we fit 1–7 profile models, each with positive parenting at age 8, children’s observed problem-solving persistence at age 8, adolescents’ self-reported inattention and impulsivity, brooding, and depression symptoms at age 14 as input variables. Although we included indicators from two ages, we did not have repeated measures across time, nor did we specify a latent transition analysis, which would examine change in profiles across time. Thus, these were not longitudinal models.

As noted, our use of Mplus version 7 [35] enables use of all available data by employing full information maximum likelihood (FIML). Percentages of missing data were low for all indicator variables (i.e., <5% missing, with the exception of the depression symptom variable, which was 72% complete). We specified a model with relatively relaxed constraints. Our model prevented cross-variable correlations within profiles but allowed all means and variances to be freely estimated, both within and across profiles. We selected the best-fitting models using three criteria: 1) the Bayesian Information Criterion (BIC), 2) entropy, and 3) utility and plausibility of profile membership and pattern.

**Fig 1:**
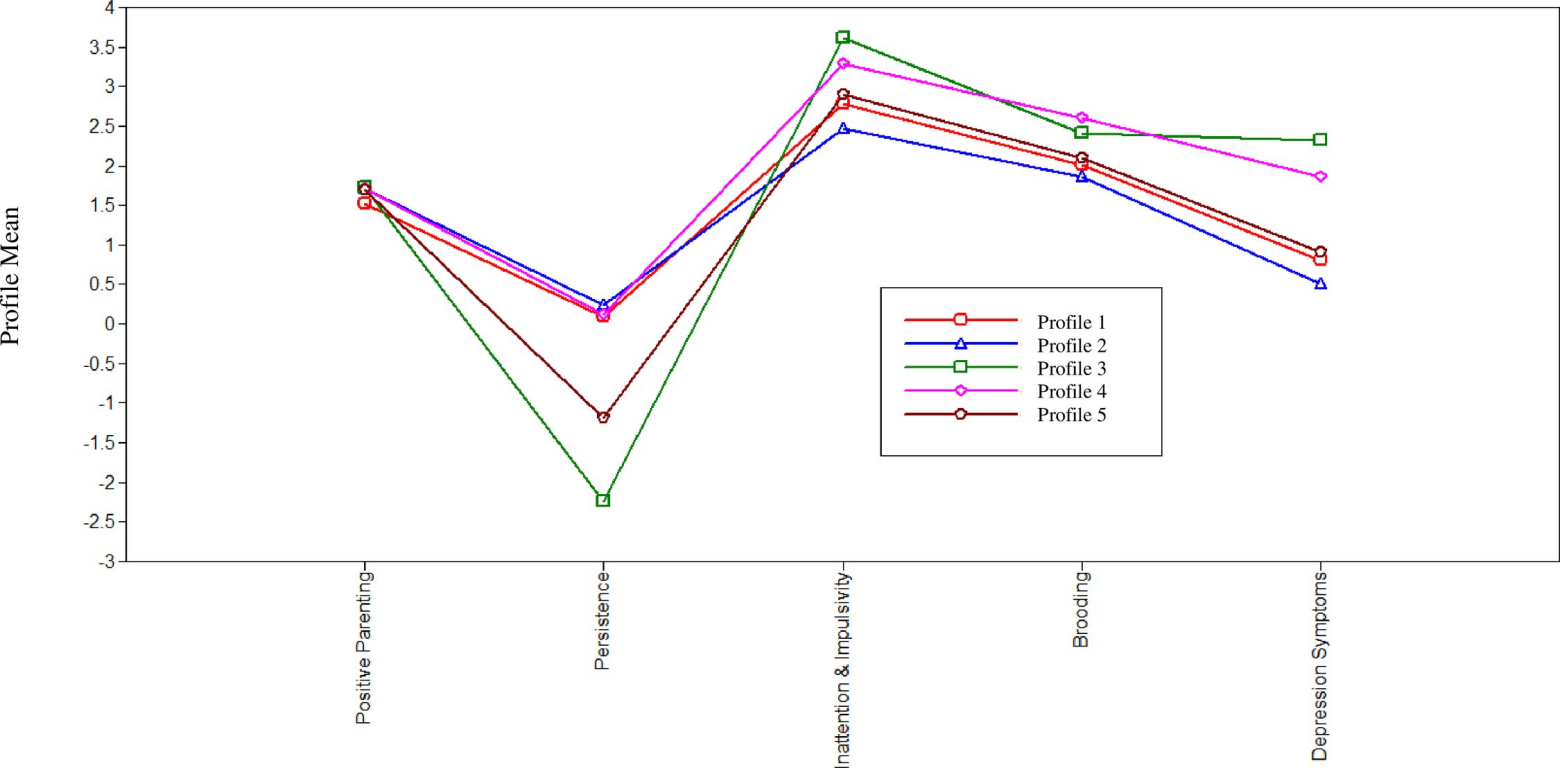
Illustration of the 5-profile solution.

Model fits for models specifying 1–7 profiles are provided in Table 2. The 6- and 7-profile models did not converge, likely due to low variance as sample sizes for profile membership got smaller. The 1- and 2-profile models have the highest BICs and lowest entropies (Table 2). Therefore, we do not examine the 1, 2, 6, or 7-profile models further. Based on the three criteria described above, the 3-, 4-, and 5-profile models all represent plausible solutions. We view the 5-profile model as best-fitting and most readily interpretable, as we describe below. Fig 1 shows relative standing on each of the indicators for the 3-, 4-, and 5- profile models.

**Table 2:** Fit indices and class membership for latent profiles.

Number of Profiles	1	2	3	4	5	6 –with errors	7 –with errors
Number of Free Parameters	12	17	24	28	**38**	45	52
Loglikelihood Value	-3,173.69	-3,193.36	-3,068.05	-3,014.39	**-2,971.41**	-2,930.50	-2,904.13
BIC	6,431.88	6,506.30	6,304.89	6,246.82	**6,210.10**	6,177.51	6,174.01
Sample Size Adjusted BIC	6,393.76	6,452.30	6,228.66	6,148.36	**6,089.40**	6,034.58	6,008.84
Entropy	n/a	.68	.76	.77	**.79**	.79	.77
Class Membership	1–1,142, 100%	1–817, 72.0%	1–113, 10.0%	1–103, 9.1%	**1–57, 5.0%**	1–150, 13.2%	1–29, 2.6%
		2–317, 28.0%	2–247, 21.8%	2–69, 6.1%	**2–694, 61.2%**	2–630, 55.6%	2–148, 13.1%
			3–774, 68.3%	3–730, 64.4%	**3–22, 1.9%**	3–38, 3.4%	3–201, 17.7%
				4–232, 20.5%	**4–232, 20.5%**	4–81, 7.1%	4–41, 3.6%
					**5–129, 11.4%**	5–22, 1.9%	5–44, 3.9%
						6–213, 18.8%	6–569, 50.2%
							7–102, 9.0%

Note: We conducted LPA analyses on data with 1 to 7 profiles. The best-fitting solution is bolded.

#### Typical profile

The *typical* profile was by far the largest group (n = 694); it comprised individuals with levels near the mean for all of our indicator variables.

#### Low persistence profiles

The 5-profile model identified both a *low childhood persistence* profile (n = 129, 11.4%) and a profile with very few members (n = 22, 1.9%) with very low childhood persistence combined with a mixture of adolescent internalizing and externalizing psychopathology symptoms, which we called the *very low childhood persistence/mixed symptoms* profile. Members of the *low childhood persistence* profile notably lacked the depression symptom and inattention/impulsivity elevations seen in the *very low childhood persistence/mixed adolescent symptomology* profile.

#### Mildly elevated typical

A profile in which participants exhibited slightly higher levels of inattention, brooding, and depression symptoms than the *typical* profile, as well as slightly lower levels of positive parenting and childhood problem solving persistence, was differentiated in the 5-profile model (n = 57, 5.0%).

#### Elevated adolescent symptomology profile

A relatively large profile (n = 232, 20.5%) emerged in which members reported mild to moderate elevations in adolescent inattention/impulsivity, brooding, and depression symptoms, but had typical levels of positive parenting and persistence.

### Profile membership and suicidal ideation endorsement

We then conducted a regression using profile membership to predict suicidal ideation endorsement. Compared with the *typical* profile, participants in the *elevated adolescent symptomology* and *very low childhood persistence/mixed symptoms* profiles were both significantly more likely to report suicidal ideation during adolescence (b_adolescent symptomology_ = 2.6, p = .001; b_childhood persistence_ = 5.3, p < .001).

We do not discuss the 3 and 4-profile models in depth because we found that the 5-profile model fits the data best. However, it is worth noting that the three- and four-profile models did not distinguish between the *low childhood persistence* and *very low childhood persistence/mixed symptoms*, which had notably discrepant associations with suicidal ideation endorsement. This suggests that the five-profile solution is more useful in identifying individuals who may report suicidal thoughts because it can offer more refined insight into how profiles with subtle differences in indicator variable means may relate differentially to suicidal ideation likelihood.

## Discussion

We used latent profile analysis to expand on traditional univariate analyses of risk factors for adolescent suicidal ideation by synthesizing a mixture of distal and concurrent cognitive, behavioral, and familial risk predictors. While traditional top-down classification of suicidal ideation uses diagnostic criteria or rating scale cutoffs to assess risk, person-centered approaches like latent profile analysis permit a complementary, empirically-derived, bottom-up approach to classifying adolescent suicidal ideation risk. Five distinct profiles emerged from our analyses: *typical*, *low childhood persistence*, *very low childhood persistence/mixed symptoms*, *mildly elevated typical*, and *elevated adolescent symptomology*. These five distinct and diverse profiles provide novel insight into how constellations of childhood and adolescent risk factors relate to likelihood of suicidal ideation endorsement during adolescence.

### Profile membership and adolescent suicidal ideation endorsement

These findings expand on results from our prior logistic regression analyses involving longitudinal behavioral predictors by clarifying how they fit in a broader picture of social and cognitive processes that occur more proximally to suicidal ideation endorsement. Specifically, although individual regression analyses revealed a significant association between reduced childhood problem-solving persistence and adolescent suicidal ideation risk [2], they were not conducive to inferences regarding how childhood behavior clusters with other risk factors that are more temporally proximal to adolescent suicidal ideation. The finding that lower childhood problem-solving persistence differentiated two profiles (i.e., *low persistence* and *very low persistence/mixed adolescent symptomology*) from the others indicates that this behavioral risk factor relates meaningfully to suicidal ideation endorsement likelihood in univariate and multivariate analyses. In addition, the finding that membership in the *elevated adolescent symptomology* profile is associated with increased likelihood (OR = 2.6, p = .001) of reporting concurrent suicidal thoughts aligns well with our prior findings that adolescent brooding, inattention, and impulsivity were associated with higher likelihood of concurrent suicidal ideation reports [5]. Thus, it is unsurprising that these variables were also the defining features of a latent profile whose members reported suicidal thoughts at a significantly elevated rate.

Compared with approaches that would only clarify the indicator variables’ relative predictive strengths within a regression model (e.g., multivariate logistic regression), our LPA findings shed light on how combinations of distal and concurrent tendencies *within a person* relate to suicidal ideation endorsement. The utility of distal predictors in our analyses is consistent with the finding of King et al. [36] that childhood experiences like physical and sexual abuse characterize numerous profiles of adolescents who present to emergency departments and are at heightened risk for suicide. Collectively, these findings highlight the value of considering distal factors in addition to prior suicidal thoughts and behaviors when examining typologies associated with concurrent suicidal thoughts and actions. Presumably, even more comprehensive assessment of adolescents’ prior experiences and functioning would further enrich future latent profile analyses and enhance their predictive power.

When combined with our previous findings regarding the relationship between lower levels of childhood problem-solving persistence and subsequent suicidal ideation [2], the *low persistence* and *very low persistence/mixed symptoms* profiles offer additional insight into characteristics that may cluster with low persistence and how they differentially relate to likelihood of suicidal ideation endorsement. The finding that markedly low childhood problem-solving persistence (i.e., *very low persistence/mixed symptoms* profile) clusters with heightened inattention/impulsivity and depression symptoms and is associated with significantly greater likelihood of reporting suicidal thoughts (OR = 5.3, p < .001) bolsters the hypothesis that early problem-solving deficits, particularly when they are pronounced, are a meaningful predictor of subsequent suicidal ideation [1]. Notably, membership in the *low childhood persistence* profile was not associated with increased likelihood of reporting suicidal ideation during adolescence, suggesting that decreased problem-solving persistence during childhood is more likely to be associated with endorsement of suicidal ideation if it is markedly, rather than mildly, low and followed by the emergence of psychopathology symptoms. This pattern would not be apparent in regression analyses and underscores an added benefit of latent profile analyses.

### Strengths of latent profile analysis

We do not contend, based on our pattern of results, that children who display very low persistence during problem-solving tasks are a categorically distinct group. However, our findings do offer useful insight into putative risk and resilience typologies. Specifically, our results suggest the likely benefit of examining biological and additional environmental influences on low persistence and its association with subsequent suicidality, even in the context of positive parenting.

### Intervention implications

If our findings are replicated, particularly in higher risk samples, they could have useful implications for designing more precise interventions. Specifically, using a person-centered, profile-based analytical approach is more conducive to individualized care that is informed by chronic patterns instead of being limited to an adolescent’s clinical state at presentation. Clarification of these multifaceted typologies could not only help with pinpointing risk factor patterns that indicate a need for intervention with varying degrees of urgency, but also facilitate provision of earlier interventions for individuals who are beginning to resemble typologies associated with significant elevations in suicidal ideation risk. Moreover, models that include non-diagnostic variables, including cognitive, behavioral, and social risk factors, provide valuable insight into novel risk factors and may shed light on previously overlooked sources of resilience that may bolster suicide prevention programs.

### Limitations & conclusions

Although latent profile analysis is useful in identifying patterns of behavior within a person, the quality of results is dependent on many factors, including, but not limited to, sample size, the number of and variation in indicator items, and differences in model specification. Additionally, latent profile analysis is not ideally suited to definitively clarify whether the reality is a heterogeneous population composed of a mixture of multiple normally distributed, homogeneous subpopulations or a single, non-normally distributed homogeneous population. Although latent profile analysis provides results in the form of seemingly distinct groups, these groups do not necessarily align with unique etiologies or clinical and/or classification implications. Consequently, latent profile analysis is less compatible with a dimensional approach and introduces some level of risk of reification; thus, results should be assessed for interpretability and consistency with theoretical conceptualizations.

With regard to our specific sample and analyses, one limitation is the lack of cross-validation of latent profiles. Effective cross-validation of profiles could include splitting a larger sample or repeating analyses in a second sample, which would be unlikely to have exactly the same set of measures. Latent profile analyses in large clinical samples would provide useful opportunities to test a range of additional hypotheses. Additionally, although not a limitation of the analyses we conducted, we only examined our profiles in relation to suicidal ideation, not suicide attempts or death by suicide, due to the low frequency of these outcomes in our sample. We also emphasize that profiles in which a relatively high proportion of members reported suicidal thoughts would not necessarily generalize to suicide attempts and/or death by suicide, as these outcomes often have unique risk factors [37, 38]. Although the diverse longitudinal measures included in our sample are a notable strength and offer insight into a wide range of risk factors for adolescent suicidal ideation, we still cannot fully exclude confounding variables, which preclude clear causal inferences. For instance, rates of endorsement of suicidal ideation in our sample are somewhat lower than those from other community-based adolescent samples [39]. This difference may be attributable to the relatively high degree of family stability in our sample. Specifically, our sample of families includes a markedly lower rate of parental divorce than is typical nationwide (i.e., 12% of participants’ biological parents reported being divorced vs. 40–50% nationally in the U.S.), and our participants came from families who had been participating in a longitudinal study since early childhood. Such participation implies a degree of family stability that may decrease the odds of suicidal ideation, especially considering findings linking low family cohesion to adolescent suicidal ideation [40]. In future replications and extensions of these findings, including more variables, as well as more reporters for each variable, would permit a more exhaustive model fitting process, which could yield additional insight into more fine-grained facets of suicidal thoughts and behaviors (*e*.*g*., grouping suicide attempts by lethality level) or profiles that were not sufficiently common for detection in our sample. Moreover, validation of the five profiles we observed should extend beyond our examination of whether endorsement of suicidal ideation was associated with significantly higher or lower probability of being placed in each profile.

These limitations notwithstanding, our approach did yield insights into how latent profile analysis can synthesize a variety of concurrent and distal suicidal ideation risk factors into typologies that could prove clinically useful, if they can be replicated and extended. Additionally, the crucial role that low and very low levels of childhood problem-solving persistence played in differentiating profiles highlights the generally overlooked benefits of utilizing longitudinal, behavioral variables to enhance prediction of adolescent suicidal ideation.
